# Smoking-associated Electrocardiographic Abnormalities Predict Cardiovascular Mortality: Insights from NHANES

**DOI:** 10.21203/rs.3.rs-3615687/v1

**Published:** 2024-01-01

**Authors:** Affan Irfan, Daniel W. Riggs, George Koromia, Andrew Paul DeFilippis, Elsayed Z. Soliman, Aruni Bhatnagar, Alex P. Carll

**Affiliations:** Mayo Clinic; University of Louisville; Marshall University; University of Louisville; Wake Forest University; University of Louisville; University of Louisville

**Keywords:** tobacco, nicotine, electrocardiogram, repolarization, conduction, ventricular

## Abstract

**Background—:**

Smoking is associated with arrhythmia and sudden cardiac death, but the biological mechanisms remain unclear. Abnormal electrocardiogram (ECG) durations of ventricular repolarization (QT interval), atrial depolarization (P wave), and atrioventricular depolarization (PR interval and segment), predict cardiac arrhythmia and mortality.

**Objectives—:**

To elucidate how smoking affects cardiac excitation, we assessed in a nationally representative sample (NHANES III) associations between cotinine, abnormalities in P duration, PR interval, PR segment, rate-corrected QT (QTc), QRS duration, and JT interval, and long-term mortality.

**Methods—:**

We analyzed data from 5,633 adults using survey-weighted multinomial logistic regression to estimate associations between tobacco use (>15 ng/ml serum cotinine) and short (<5th percentile) or long (>95th percentile) ECG intervals, relative to reference (5 – 95th percentile).

**Results—:**

After adjustment for demographics, risk factors, and conduction-altering medications, smoking was associated with a higher odds of short PR interval, PR segment, and QRS, and long JT. Broader ECG effects of smoking were also assessed by survey-weighted linear regression of continuous cotinine and ECG intervals, which revealed cotinine inversely associated with PR segment and QTc. Over a 22-year follow-up, many ECG abnormalities predicted cardiovascular mortality in smokers, including long JT, QRS, and QTc, and short QRS.

**Conclusions—:**

Smoking increases likelihood for rapid atrioventricular conduction, rapid ventricular depolarization, and slow ventricular repolarization. The ventricular electrophysiologic abnormalities associated with smoking also predict cardiovascular mortality in smokers; however, traditional ECG measures of cardiac risk like QTc can overlook these ventricular defects and their independent predictive value in smokers.

## Introduction

Smoking is the leading preventable cause of cardiovascular morbidity and mortality attributable to coronary artery disease and cardiac arrhythmias.^1, 2^Smoking is associated with a 2-fold higher risk of atrial fibrillation (AF)^3^and is estimated to cause 1 of every 15 AF-related deaths among men.^4^ Given that smoking is associated with arrhythmia prevalence in a dose-dependent manner,^5^ we hypothesized that the intensity of smoking corresponds with pro-arrhythmic changes in cardiac electrical function. Consistent with this hypothesis, we recently reported that in a small outpatient cohort of 136 patients smoking was associated with faster atrioventricular (AV) conduction in conjunction with higher urinary dopamine levels and at magnitudes predictive of adverse clinical outcomes.^6^ In the present study we tested for associations between smoking and cardiac electrical abnormalities in a large, nationally representative cohort, the Third National Health and Nutrition Examination Survey (NHANES-III), and evaluated the association of these abnormalities with long-term cardiovascular and all-cause mortality.

The mechanisms underlying the arrhythmogenic effects of smoking remain unknown. Although tobacco smoke contains a wide range of constituents that can affect cardiac excitation, nicotine may be an important trigger. Extensive work has shown that nicotine increases sympathetic neural activity and circulating catecholamines,^7^ and that it induces oxidative stress and fibrosis^7, 8^ and inhibits potassium channels involved in ventricular repolarization.^9^ The recent increase in use of electronic nicotine delivery systems (ENDS), and our findings that ENDS alter repolarization and evoke spontaneous arrhythmias in mice,^10^ lend urgency to understanding the effects of conventional smoking on cardiac electrophysiology, Electrocardiogram (ECG) abnormalities can denote defects in the conduction cycle and an increased arrhythmia risk. Both prolonged and shortened ECG indices, including the P wave,^11^ PR interval,^12^ and QT interval,^13^ are associated with cardiovascular morbidity and mortality. Moreover, abnormal values in the subcomponents of PR^14–16^ and QT intervals^17, 18^ have recently demonstrated prognostic utility

In multiple attempts to characterize the electrophysiologic effects of smoking, previous studies assessed resting 12-lead electrocardiograms (ECGs) in smokers.^19^ In NHANES-III, serum cotinine and digital ECG data provide a unique opportunity to precisely examine the electrophysiological effects of smoking in a large nationally representative population. To resolve the inconsistent reports on the association between smoking and ECG, and address the limitations of these past studies (including small sample size, neglect of PR and QT subcomponents, failure to account for key covariates, crude approximations of cigarette smoking intensity, and imprecise estimates of the role of nicotine), we assessed the association between conventional smoking, confirmed by serum cotinine levels, with ECG indices and their components. We further evaluated the association of ECG abnormalities in smokers with long-term cardiovascular and all-cause mortality.

## Methods

### Study Participants

The NHANES, a periodic survey of a representative sample of the civilian un-institutionalized United States population, aims to provide estimates of disease prevalence and the overall health status of the population. All participants gave a written informed consent at the time of the survey. Baseline data were collected from 1988 to 1994 during an in-home interview (demographics, past medical history including smoking status, and use of medications) and subsequent visit to a mobile examination center (ECG, blood samples, and basic laboratory values for serum cotinine, total cholesterol, high-density lipoprotein cholesterol, and plasma glucose). Serum cotinine was used to categorize participants as smokers (> 15 ng/ml), or nonsmokers (≤ 15 ng/ml).^20^ We included in analyses all participants with clean ECG recordings, no major ECG abnormalities (including evidence of myocardial infarction or ischemia as defined by Minnesota Electrocardiogram Classification), and corresponding data for serum cotinine, medical history, medication use, and anthropometric measurements. See the Supplement for further details on criteria for patient characteristics. Participants were linked with the public-use linked mortality file from the National Center for Health Statistics, (https://www.cdc.gov/nchs/data-linkage/mortality-public.htm). The mortality status of the population was ascertained by the National Death Index through December 31, 2015. Cause specific death was coded by ICD-10. The primary outcomes for our analysis were mortality from all causes, and diseases of the heart (ICD-10 codes: I00-I09, I11, I13, I20-I51, or I60-I69).

### ECG Data Acquisition and Analyses

Standard 12-lead ECGs were recorded using a Marquette MAC 12 system (Marquette Medical Systems, Milwaukee, Wisconsin) by trained technicians. Computerized automated analysis of the electrocardiographic data was performed with visual inspection of outlier values by a trained technician in a central ECG core laboratory (EPICARE Center at the Wake Forest School of Medicine, Winston Salem, NC). PR interval and P duration in lead II and global QRS duration and QT interval were automatically measured, and with subsequent calculations for JT (= QT – QRS) and PR segment (= PR interval – P duration). QT interval was heart rate corrected using the Framingham formula (QTc), calculated as QT + 154 × (1 − 60/HR).^21^ To measure the association between cotinine and abnormal ECG intervals, we stratified participants into three groups for each given ECG index: >95th percentile (long), 5–95th percentile (reference) and < 5th percentile (short) (Table S1).

### Statistical Analysis

ECG sampling weights were used in the analysis to account for the complex sampling design^22^ and ensure generalizability of results to the national population. Categorical variables were reported as frequency and population percentages, whereas continuous variables were recorded as geometric mean ± standard error for all demographic tables. Statistical significance in demographic tables was determined using survey-weighted analysis. Student’s *t* test or ANOVA were performed for continuous variables, whereas Rao-Scott chi-square was used for categorical variables. Survey-weighted multinomial regression was used to calculate the odds ratios and 95% confidence intervals for the association between serum cotinine levels (> 15 ng/ml) and ECG intervals using the median group (5–95th percentile) as reference. Sensitivity analysis was performed, excluding participants with serum cotinine > 15 ng/ml but self-reported as never-smokers (n = 134) or ex-smokers (n = 216).

Multivariable adjusted models were constructed with incremental adjustments as follows: model 1: adjusted for demographics including age, sex and race-ethnicity; model 2: adjusted for model 1 covariates, heart rate (excluded for QTc), and cardiovascular risk factors, including obesity, diabetes, hypertension, dyslipidemia, previous cardiovascular disease, congestive heart failure, chronic obstructive pulmonary disease and alcohol intake; and model 3: adjusted for model 2 covariates, and select medications, including beta blockers, calcium channel blockers and anti-arrhythmic drugs. Model 4 was adjusted for baseline variables which significantly differed (P < 0.10) between groups for a given ECG parameter (Tables S2-S7), including those specifically for PR interval (age, sex, previous CVD, beta blockers, calcium channel blockers, heart rate), P duration (age, sex, race-ethnicity, congestive heart failure, alcohol intake, beta blockers, calcium channel blockers, heart rate), PR segment (age, sex, alcohol intake, calcium channel blockers, heart rate), QTc interval (age, chronic obstructive pulmonary disease, beta blockers, calcium channel blockers), QRS duration (sex, obese, chronic obstructive pulmonary disease, heart rate), and uncorrected JT interval (age, sex, dyslipidemia, alcohol intake, heart rate, anti-arrhythmic drugs). We also performed fully adjusted survey-weighted linear regression between continuous cotinine levels and ECG intervals as continuous variables. Subgroup analyses were performed with stratification by age (cut-off point by median − 59 years) and gender. A 2-sided P-value of < 0.05 was considered significant for main effects and for interactions.

We fit Cox proportional hazard models to compute hazard ratios (HR) and 95% confidence intervals for associations between ECG interval groups and mortality. Survival time was calculated as the number of months between NHANES interview date and death. Censored survival time was calculated as the difference between interview data and end of follow-up (December 31, 2015) or loss to follow-up. Models were adjusted as per model 3 from our cross-sectional analysis. The Cox proportional hazards models were stratified by smoking status to assess effect modification. Data were analyzed using the survey procedures in SAS, version 9.4 (SAS Institute, Inc., Cary, North Carolina).

## Results

A total of 5,633 study participants (mean age 59 ± 13 years, 53% women, 48% non-Hispanic white) were included in this analysis, including 1,580 (28%) identified as smokers (serum cotinine levels > 15 ng/ml). Smokers were younger, disproportionately male, and had lower prevalence of dyslipidemia and β-blocker use, higher chronic obstructive pulmonary disease (COPD) prevalence, resting heart rate, and alcohol intake (Table 1). Table 1 and Tables S1-S7 provide geometric means for all continuous variables and population percentages for categorical baseline characteristics.

### Supraventricular Electrophysiology.

**Figure 1:**
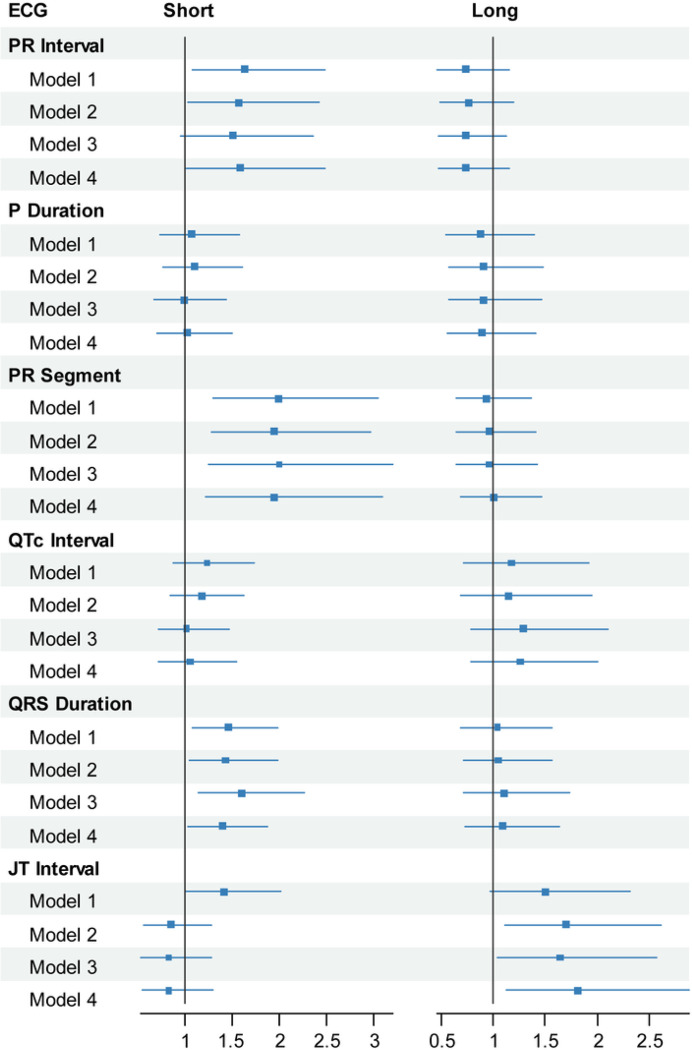
Association of ECG abnormalities with smoking status. Forest plots of hazard ratios (± 95% confidence intervals) among smokers. ECG variable groups: Short (<5^th^ percentile), reference (5–95^th^ percentile) and Long (>95^th^ percentile). Models adjusted for age, sex, race-ethnicity (Model 1), plus heart rate, obesity, diabetes, hypertension, dyslipidemia, previous cardiovascular disease, congestive heart failure, chronic obstructive pulmonary disease, and alcohol intake (Model 2), plus beta blockers, calcium channel blockers and anti-arrhythmic drugs (Model 3). Heart rate was excluded in models for QTc. Model 4 was adjusted only for those baseline variables specifically associated with a given ECG parameter in this population as described in the Statistical Analysis section of the Methods.

**Figure 2:**
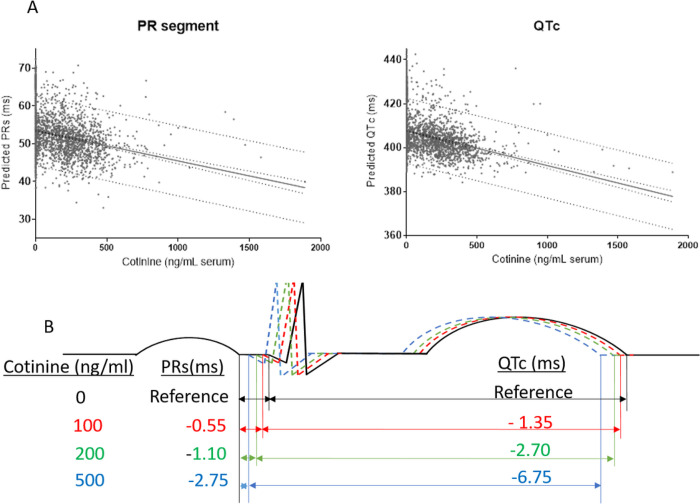
Concentration-dependent relationship between serum cotinine and shortening of PR segment or QTc. **A,** linear relationships of PR segment (ms) and QTc interval (ms) with cotinine levels. **B**, representation of progressive shortening of PR segment and QTc with corresponding increases in serum cotinine.

In multinomial logistic regression models, smoking was associated with higher odds of abnormally short PR interval (odds ratio [OR]: 1.63, 95% CI: 1.07, 2.48) and abnormally short PR segment (OR: 1.99, 95% CI: 1.30, 3.05) (Fig. 1; Table S8) after adjustment for demographics. This association was not attenuated after further adjustment for known CVD risk factors, heart rate, and confounding medications (Fig. 1), and was consistent in subgroups stratified by age or sex (Table S9). Smoking status was not significantly associated with P wave duration. Linear regression analysis revealed a significant inverse association of continuous cotinine levels with PR segment (Fig. 2; Table 2), but not with P wave or PR interval. When adjusting only for participant characteristics associated with baseline PR segment, PR segment shortened by 0.554 ms per 100-ng/ml in cotinine (Table 2).

### Ventricular Electrophysiology.

In multinomial logistic regression models adjusting for demographics, CVD risk factors, heart rate (omitted for QTc) and medications, smoking was associated with shortened QRS duration (OR: 1.60, 95% CI: 1.13, 2.26) and long JT (OR: 1.64, 95% CI 1.04, 2.57), but not with abnormal QTc (Fig. 1; Table S10). The associations of smoking status with short QRS (< 5th percentile) and long JT (> 95th percentile) remained consistent when stratifying by age or sex (Table S9). However, in linear regression models, continuous cotinine levels were not associated with QRS duration or JT interval despite consistent inverse associations with QTc across all models (Table 2; Fig. 2). In the multinomial logistic regression adjusted solely for associated baseline characteristics, QTc shortened by −1.345 ms with every 100-ng/ml increase in cotinine levels (Table 2).

### Survival analyses.

**Figure 3:**
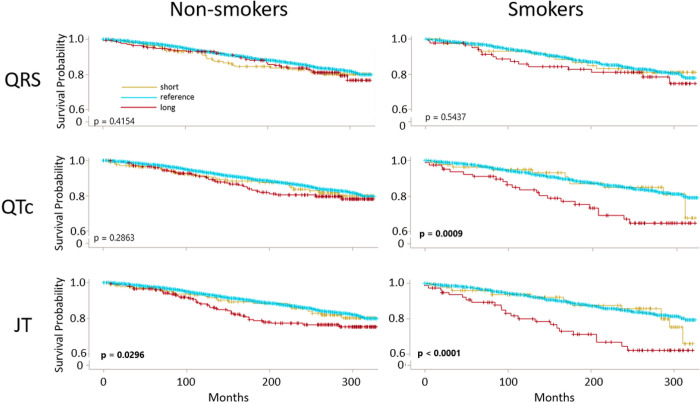
Survival curves for cardiovascular mortality by ECG abnormalities among smokers and non-smokers. ECG components were grouped by short (<5^th^ percentile), normal (5^th^-95^th^ percentile), and long (> 95^th^ percentile), Kaplan-Meier survival estimates for cardiovascular disease-attributable mortality stratified by smoking status with log-rank test p-values for differences between the three survival curves. Duration of survival is measured as months from exam.

**Figure 4:**
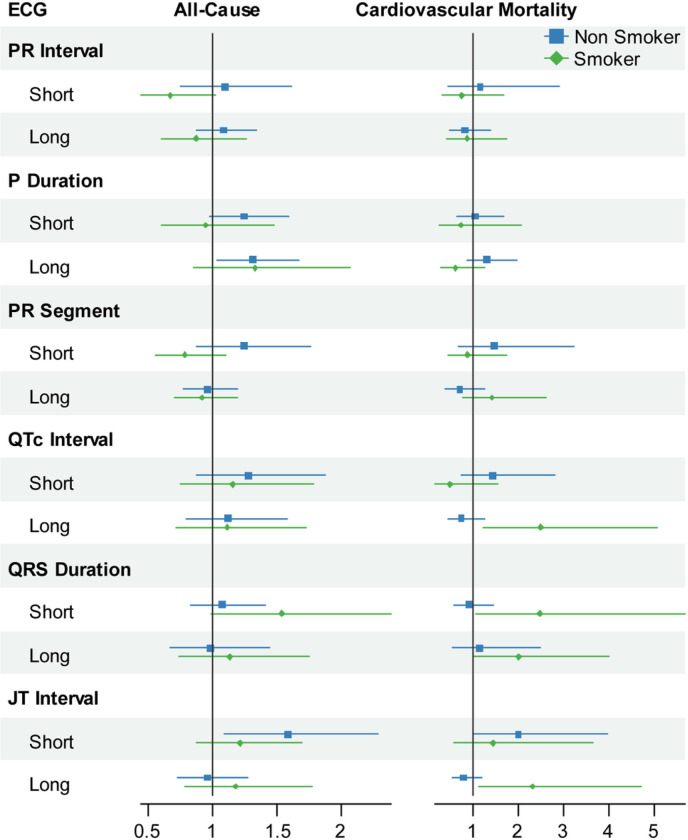
Risk of long-term mortality among smokers and nonsmokers with prior ECG abnormalities. Forest plots hazard ratios (± 95% confidence intervals) for ECG categories and mortality in smokers (green diamonds) and non-smokers (blue squares). Models adjusted for demographic variables, cardiovascular risk factors, heart rate (omitted for QTc), and select medications using covariates for Model 3 detailed in Fig 1.

To assess whether our findings could relate to mortality risk, we tested for relationships between abnormal ECG values and mortality stratified by smoking status at time of examination. Among the 1,580 participants classified as smokers, a total of 939 deaths occurred (241 from heart diseases) during a median follow-up of 21.67 years. Among the 4,073 non-smokers, a total of 2,004 deaths occurred (610 from heart disease). Crude survival estimates among smokers suggested long QRS, long JT, and long QTc associated with higher cardiovascular mortality (Fig. 3). Adjustment for confounders in Cox proportional hazard models confirmed these observations, with risk of cardiovascular mortality significantly higher for smokers with abnormally long JT (hazard ratio [HR]: 2.31, 95% CI: 1.13, 4.71), long QTc (HR: 2.50, 95% CI: 1.23, 5.06), and long QRS (HR: 2.01, 95% CI: 1.01, 4.02), as well as abnormally short QRS (HR: 2.47, 95% CI: 1.08, 5.7) (Fig. 4; Table S11). All-cause mortality was not associated with ECG abnormalities among smokers. In contrast, among non-smokers, *short* JT was associated with higher cardiovascular mortality (HR: 2.00, 95% CI: 1.01, 3.97) and higher all-cause mortality (HR: 1.58, 95% CI: 1.10, 2.29) (Table S12).

## Discussion

**Figure 5:**
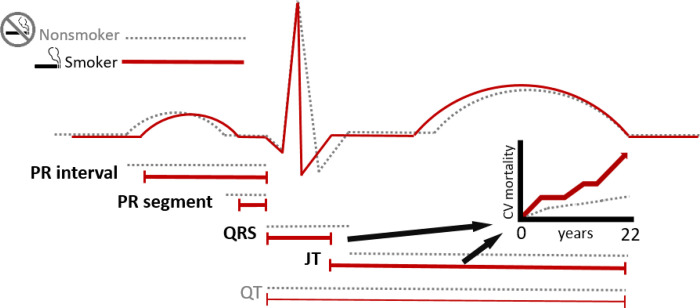
Central Illustration. Smoking in NHANES III was associated with abnormally short PR interval, PR segment, and QRS, as well as abnormally long JT interval. Short QRS and long JT each predicted higher long-term cardiovascular mortality in smokers.

In a large representative sample of the general US population, we found that smokers had significantly different baseline ECG and that smoking (as measured by cotinine) was associated with abnormally short PR interval, PR segment, and QRS duration, as well as abnormally long JT interval (Fig. 2). Among these abnormalities, short QRS and long JT were also predictive of cardiovascular mortality in smokers over a 22-year follow-up, suggesting that the effects we observed of smoking on ventricular depolarization and repolarization correspond with increased risk of cardiovascular death (Fig. 5). The positive associations of smoking with both long JT and short QRS, and of long JT and short QRS with cardiovascular mortality exclusively in smokers, jointly suggest that both rapid depolarization and slow repolarization in the ventricles predict, and may even underlie, cardiovascular mortality in smokers. Although smoking status was not associated with abnormal QTc, serum cotinine was inversely associated with QTc, as well as with PR segment. These findings suggest that smoking alters both atrioventricular and ventricular conduction and add biological plausibility to prior reports of associations of smoking with sudden death, arrhythmia, stroke, and other cardiovascular outcomes related to cardiac electrophysiological dysfunction.^1, 2^ Our observations also demonstrate that smoking accelerates the subcomponents of PR and QT that denote atrioventricular conduction and ventricular depolarization, respectively, while it slows the QT subcomponent that indicates repolarization, in a large nationally representative cohort, suggesting these emerging ECG indices can provide unique insight into the mechanisms of smoking-induced arrhythmia and adverse cardiac outcomes.

We recently revealed in a small cohort study an inverse association between cotinine and both PR interval and PR segment, with evidence that nicotine may shorten both via increased circulating dopamine.^6^ In the current study, smoking corresponded with differences in atrioventricular conduction, as measured from the PR interval and PR segment. Thus, our present observations in a large nationally representative cohort corroborate our previous findings that smoking shortens PR. Although prior studies have yielded widely varying estimates of effects of smoking on PR interval and its components,^23–25^ studies with larger sample sizes have found associations between smoking and shortened PR interval,^26–28^ consistent with our current and prior findings involving tobacco products,^6, 10^ including both chronic smokers and mice exposed acutely to ENDS aerosols. We did not find any significant association between P wave duration and cotinine levels in accordance with some studies^8, 25^ but conflicting with others as well.^29^ Thus, our null observations strengthen extant data suggesting that smoking may not overtly alter the duration of atrial depolarization despite its association with atrial fibrillation.^3^ These findings are particularly salient amidst emerging data demonstrating the ability of short PR interval to predict atrial fibrillation^12, 16^ and cardiovascular mortality.^14, 30, 31^ Our collective findings indicate that exposure to cigarette smoke, and perhaps nicotine specifically, accelerates atrioventricular conduction – which is known to elevate the risk of atrial fibrillation, heart failure, and stroke

We also found that smoking was associated with altered measures of ventricular depolarization (QRS) and repolarization (JT and QTc). Collectively, these findings suggest that prior inconsistencies in associations between smoking and QTc^32–36^ might relate to this parameter comprising both ventricular depolarization and repolarization.^37^ Consequently, assessments solely of QTc may neglect the divergent impacts of smoking on the durations of ventricular depolarization (short QRS) and repolarization (long JT) that we report here, and thus promote false conclusions that smoking does not impact repolarization. Notably, our observations that short QRS and long JT both predict cardiovascular mortality in smokers—but not in non-smokers—suggest that smoking modifies the effects of these abnormalities on mortality and may concurrently undermine the prognostic value of QT. To our knowledge, no prior studies have explored the relationship between JT interval and smoking. In addition to our recent study in a small urban cohort,^6^ only one other study has reported the effect of cotinine-measured smoking on QT interval. Similar to the current study, Zhang et al.^13^ used the NHANES III database, but—in contrast with our observations—found no association between QT interval and self-reported smoking and cotinine levels in fully-adjusted models among 7795 men and women. The discrepancy of this prior null observation from our own positive findings may be due to the authors using QTc quartiles instead of continuous QTc values. In addition, we demonstrate an association of smoking (> 15 ng/ml cotinine) with abnormally short QRS duration and abnormally long JT interval despite neither index having a linear relationship with cotinine. While seemingly incongruous, our findings that cotinine inversely correlates with QTc but smoking increases the odds of abnormally long JT accord with continuous cotinine representing real-time nicotine levels and sympathetic acceleration of repolarization, and a cotinine threshold better delineating smoking status and risk for an extreme repolarization abnormality. Importantly, prolonged JT interval is a known predictor of mortality in the general population.^17, 18^ The present observations also identify abnormally prolonged JT as a predictor of long-term cardiovascular mortality in smokers. Although prolonged QRS duration has been shown to predict mortality in the general population^38^, the current data demonstrate that both abnormally short and abnormally long QRS duration predict long-term cardiovascular mortality among smokers.

Given the sympathomimetic effects of nicotine, autonomic imbalance is a plausible mediator of our observations, which can accelerate AV conduction and ventricular depolarization and delay ventricular repolarization. Indeed, sympathetic excitation may mediate the shortening of QRS (ventricular depolarization)^39, 40^ and prolongation of JT (ventricular repolarization)^41–43^ similar to our recent evidence that smoking shortens AV nodal conduction via nicotine and catecholamines.^6^ Although cellular-level and autonomic mechanisms exceed the scope of our present study, it is noteworthy that our findings support a link between smoking and cardiac ion channels while further implicating nicotine and identifying additional impacts on ventricular depolarization and repolarization in a nationally representative population. Despite our inability to distinguish between effects of nicotine vs. other constituents in cigarette smoke, our current observations complement recent findings suggesting that e-cigarettes may also alter electrophysiology in a nicotine-dependent manner.^44^

Despite its numerous strengths, the study also has some limitations. First, a single measure of serum cotinine may imprecisely reflect chronic smoking, as cotinine directly indicates recent nicotine exposure with a half-life of 16–19 h. Nonetheless, studies have demonstrated that a single measure of cotinine can be used to represent chronic smoking intensity and nicotine intake^20, 45^ as smokers tend to maintain constant nicotine levels. Moreover, because nicotine levels strongly correlate with other harmful or potentially harmful substances in cigarettes (such as tar or volatile organic compounds),^46^ it seems reasonable to infer exposure intensity for other cigarette constituents (e.g., tar) from cotinine. In addition, even though we adjusted for several potential confounders, we recognize the possibility of residual confounding due to variables not accounted for in the study. Further, our analysis between smoking status and ECG parameters is cross-sectional, and thus we cannot rule out reverse causality. Despite these limitations, our data provide seminal evidence in a nationally representative cohort that smoking may increase risk for cardiovascular mortality by causing aberrant ventricular depolarization and repolarization.

## Conclusions

We found in this large representative sample of the U.S. population that elevated serum cotinine levels are independently associated with abnormally fast atrioventricular conduction (short PR), and ventricular depolarization (short QRS), and abnormally slow ventricular repolarization (long JT). Some of these abnormalities in smokers (short QRS and long JT) were associated with higher cardiovascular mortality risk. Additionally, increasing cotinine was associated with progressive shortening of PR segment and QTc when considering the normal bounds of these parameters. Collectively, our findings indicate that smoking may progressively modify regular cardiac conduction while also increasing risk for electrophysiologic abnormalities that specifically predict long-term cardiovascular mortality.

## Figures and Tables

**Table 1:** Baseline Participants Characteristics (Total N = 5,653)

Characteristic	Smoker		p-value
	No ≤ 15 ng/ml n = 4073, 72%	Yes >15 ng/ml n = 1580, 28%	
Age (years)*	56.4 ± 0.44	53.4 ± 0.45	<0.001
Women	2371 (59.1%)	629 (43.7%)	<0.001
Non-Hispanic White	2122 (82.2%)	738 (80.8%)	0.291
Smoking status			<0.001
Never	2381 (56.1%)	134 (5.8%)	
Current	67 (1.6%)	1230 (78.4%)	
Past	1625 (42.3%)	216 (15.8%)	
Diabetes mellitus	479 (7.5%)	143 (7.0%)	0.630
Hypertension	1427 (31.8%)	489 (28.8%)	0.167
Dyslipidemia	1066 (29.3%)	308 (23.1%)	0.004
Obesity	853 (18.0%)	233 (15.3%)	0.120
Chronic Obstructive Pulmonary Disease	263 (6.8%)	160 (10.8%)	<0.001
Heart rate (beats/minute)	67.6 ± 0.3	68.9 ± .04	0.012
Prior cardiovascular disease	172 (3.2%)	67 (3.5%)	0.698
Congestive Heart Failure	131 (1.8%)	54 (1.7%)	0.872
Alcohol drinks per month			
0	2415 (50.7%)	762 (45.1%)	0.003
1–4	670 (18.0%)	253 (17.2%)	
5–13	440 (13.9%)	200 (13.3%)	
>13	539 (17.4%)	359 (24.4%)	
Beta blockers	320 (8.2%)	87 (5.9%)	0.009
Calcium channel blockers	333 (6.7%)	103 (6.0%)	0.453
Antiarrhythmic drugs	34 (0.6%)	17 (1.5%)	0.129

* Except for age (which is represented by geometric mean and standard deviation), all other variables are represented as frequency and column percentages.

**Table 2:** Adjusted survey weighted linear regression between serum cotinine levels and ECG intervals and their components.

Model	PR interval (ms)		P duration (ms)		PR segment (ms)	
	β (95% CI)	p-value	β (95% CI)	p-value	β (95% CI)	p-value
1 *	−0.832 (−1.549, −0.116)	0.024	−0.078 (−0.444, 0.288)	0.671	**−0.755 (−1.355, −0.154)**	**0.015**
2^†^	−0.593 (−1.320, 0.134)	0.107	−0.036 (−0.415, 0.343)	0.849	−0.557 (−1.133, 0.019)	0.058
3^	−0.542 (−1.262, 0.179)	0.137	0.036 (−0.362, 0.434)	0.856	**−0.578 (−1.135, −0.021)**	**0.042**
4^Δ^	−0.603 (−1.301, 0.094)	0.089	−0.008 (−0.391, 0.374)	0.965	**−0.554 (−1.090, −0.018)**	**0.043**
	Corrected QT (ms)		QRS duration (ms)		JT interval (ms)	
1 *	**−1.448 (−2.375, −0.521)**	**0.003**	**−0.251 (−0.489, −0.013)**	**0.039**	**−1.198 (−2.043, −0.354)**	**0.006**
2^†^	**−1.402 (−2.347, −0.456)**	**0.005**	−0.184 (−0.414, 0.045)	0.113	0.130 (−0.327, 0.587)	0.570
3^	**−1.112 (−2.056, −0.169)**	**0.022**	−0.194 (−0.433, 0.044)	0.108	0.189 (−0.300, 0.679)	0.441
4^Δ^	**−1.345 (−2.269, −0.421)**	**0.005**	−0.139 (−0.363, 0.086)	0.221	0.233 (−0.272, 0.738)	0.358

* Adjusted for demographics (age, sex and race-ethnicity)

† Adjusted per model 1 plus cardiovascular risk factors (obesity, diabetes, hypertension dyslipidemia, previous cardiovascular disease, congestive heart failure, chronic obstructive pulmonary disease and alcohol intake) and heart rate (omitted for QTc)

^ Adjusted per model 2 plus selected cardiac medications (beta blockers, calcium channel blockers and anti-arrhythmic drugs)

Δ Adjusted for baseline variables found significantly associated with ECG intervals as described in Fig. 1.

## Data Availability

The datasets used and/or analysed during the current study available from the corresponding author on reasonable request.

## Abbreviations

AF: atrial fibrillation
AV: atrioventricular
CI: confidence interval
COPD: chronic obstructive pulmonary disease
CVD: cardiovascular disease
ENDS: electronic nicotine delivery systems
HR: hazard ratio
NHANES III: Third National Health and Nutrition Examination Survey
QTc: rate—corrected QT interval
